# Molecular Mechanisms of Memory Consolidation That Operate During Sleep

**DOI:** 10.3389/fnmol.2021.767384

**Published:** 2021-11-18

**Authors:** Irene Reyes-Resina, Sebastian Samer, Michael R. Kreutz, Anja M. Oelschlegel

**Affiliations:** ^1^Research Group Neuroplasticity, Leibniz Institute for Neurobiology, Magdeburg, Germany; ^2^Leibniz Group ’Dendritic Organelles and Synaptic Function’, Center for Molecular Neurobiology, ZMNH, University Medical Center Hamburg-Eppendorf, Hamburg, Germany; ^3^Center for Behavioral Brain Sciences, Otto von Guericke University, Magdeburg, Germany; ^4^German Center for Neurodegenerative Diseases (DZNE), Magdeburg, Germany

**Keywords:** sleep, memory consolidation, synaptic plasticity, synaptic scaling, immediate early genes

## Abstract

The role of sleep for brain function has been in the focus of interest for many years. It is now firmly established that sleep and the corresponding brain activity is of central importance for memory consolidation. Less clear are the underlying molecular mechanisms and their specific contribution to the formation of long-term memory. In this review, we summarize the current knowledge of such mechanisms and we discuss the several unknowns that hinder a deeper appreciation of how molecular mechanisms of memory consolidation during sleep impact synaptic function and engram formation.

## Introduction

Considered to be downtime, away from external input, sleep opens a time window for synaptic maintenance to ensure homeostasis and plasticity (Tononi and Cirelli, 2006, 2014; Niethard et al., 2017; Turrigiano, 2017), and for the consolidation of long-term memories (Graves et al., 2001; Maquet, 2001; Stickgold et al., 2001; Fischer et al., 2002; Walker et al., 2002; Peigneux et al., 2004; Walker and Stickgold, 2004; Westerberg et al., 2012). Sleep is divided into two alternating phases, REM (rapid eye movement) and NREM (non-REM) sleep. Both occur in repeating cycles, with NREM always preceding REM sleep. REM sleep is characterized by vivid dreams, muscle atonia, brought about by the inhibition of motor neurons, and a paradoxical, wake-like electroencephalogram (EEG), with a predominance of theta activity (4/5–7/8 Hz). In humans, NREM sleep has been differentiated into four or more recently three stages (Moser et al., 2009). The first two stages represent light sleep, while the last two NREM stages, nowadays combined into one, represent deep or slow-wave sleep (SWS) with a predominance of delta-waves (human 1–4 Hz, rodents 2–4 Hz; high amplitude, but lower than slow oscillations and more local) and slow oscillations (SO, 0.5–1 Hz, higher amplitude and more global) as “separate classes of slow-waves” (Kim et al., 2019). The precise temporal coordination of cortical slow oscillations, delta waves, thalamic spindles (humans 12–16 Hz, rodents 9–15 Hz), and hippocampal sharp wave ripples (extracellular negative waves followed by fast ripples, ripples 140–220 Hz) is considered a key driver for the process of long-term memory consolidation and forgetting (Latchoumane et al., 2017; Kim et al., 2019; Klinzing et al., 2019; Langille, 2019; Ngo and Born, 2019). This division into NREM stages is usually not applied to rodents, even though it was suggested that this could be useful with regards to the understanding of temporal proceedings and underlying mechanisms of memory consolidation (Genzel et al., 2014; Luo and Guan, 2018). It is still a matter of debate which role the distinct sleep phases or sleep states play in maintaining synaptic plasticity and in consolidating previously encoded information.

Several hypotheses have been presented of how sleep might promote memory consolidation at the systemic, synaptic, and molecular level, all of them presumably converging on a scenario where the brain has to be “reset” on a global level, while selected memory traces are refined and strengthened to consolidate what is to be preserved. We will focus on the cellular and synaptic level of memory consolidation as well as on the molecular processes underlying it. To this end, we will introduce synaptic scaling and intrinsic plasticity as two synergistic mechanisms to ensure homeostasis and maintain synaptic plasticity, and with that the ability to learn and memorize new information. We review briefly the structural and electrophysiological evidence that was found for synaptic plasticity during sleep in animals that might or might not have been subjected to behavioral tasks. Subsequently, we will focus on molecular mechanisms in support of sleep-dependent memory consolidation. Here, we demonstrate gene expression patterns across the sleep-wake cycle and review oscillating kinase activity found to facilitate increased synaptic potentiation during wakefulness and depression during sleep. We discuss the hypotheses that enhanced neuronal activity and learning during wakefulness might prime neurons for processing during sleep and that immediate early genes (IEGs) might drive plasticity and would thus be critical for memory consolidation before concluding with findings about increasing IEG expression in primed neurons during sleep to promote long-term memory consolidation.

### Memory Formation—Encoding During Wake and Consolidation During Sleep

During wakefulness, the nervous system is processing a plethora of incoming information. Preserving memory for some of them involves different brain regions and retention may vary greatly in time. Here, we are reviewing the consolidation of long-term memory from transient memory traces that are sensitive to interference into persistent memory traces that are insensitive to interference until recalled again. Long-term memory is divided into declarative and procedural memories. Both involve different brain regions. Declarative memory storage involves neocortical structures as well as structures of the medial temporal lobe (e.g., Squire et al., 2004; Squire and Wixted, 2011). Contrarily, procedural memories employ the striatum and cerebellum in addition to neocortical structures (Doyon et al., 2003), even though the hippocampus is still involved in the initial stages of procedural task learning (Poldrack et al., 2001). There is strong evidence that sleep promotes the consolidation of both kinds of memory—including putative scenarios for systems consolidation (Diekelmann et al., 2009). Historically, many studies that examined the role of sleep for long-term memory consolidation, especially when working with rodents, looked into declarative, episodic memory and here indeed mostly into spatial (track running, Morris-water-maze, object-place recognition) or contextual memory (conditioned fear memory). The active systems consolidation hypothesis suggests a scenario in which the “corticalization” of declarative memories would result in the extraction of gist-like (semantic) memory representations than to be integrated into existing cortical memory representations. Other studies, especially those exploring structural changes in the context of sleep promoted memory consolidation, also looked at procedural memories (motor learning, visual cortex plasticity), where the high cholinergic activity during REM seems to be particularly crucial for successful consolidation (Rasch et al., 2009).

#### Encoding During Wake

Synaptic plasticity has been proposed to play a central role in the capacity of the brain to incorporate transient experiences into persistent memory traces, so-called engrams. Among the different types of synaptic plasticity, mainly Hebbian and homeostatic types of plasticity are likely relevant for molecular mechanisms of memory consolidation (see “Glossary” section). The encoding of information takes place during wake and results in stimulus-induced synapse-specific modification of synaptic strength and cell-specific changes of neuronal excitability (Zhang and Linden, 2003; Lisman et al., 2018). Events are presumably encoded in an assembly of neurons (engram cells) that are activated during the event and here, in turn, in a set of selected synapses (Liu et al., 2012; Ramirez et al., 2013; Cowansage et al., 2014; Denny et al., 2014; Nabavi et al., 2014; Redondo et al., 2014; Tanaka et al., 2014; Hayashi-Takagi et al., 2015; Josselyn et al., 2015; Ryan et al., 2015; Tonegawa et al., 2015, 2018; Holtmaat and Caroni, 2016; Andersen et al., 2017; Hoshiba et al., 2017; Choi et al., 2018; Clawson et al., 2021). It was suggested that the fine-tuned modification in synaptic weight could be a mechanism to determine the specific neuronal circuit that represents the encoded information and would be employed for memory retrieval. Activation of certain signaling pathways and molecular reorganization of spine synapses (e.g., actin polymerization, incorporation or removal of AMPA-receptors into the postsynaptic membrane) can either lead to synaptic potentiation or depression according to changes in dendritic spine morphology, depending on the characteristics of synaptic activation (Watt and Desai, 2010). Maintenance of such plastic changes in synaptic strength and neuronal connectivity essentially requires *de novo* protein synthesis and gene expression. An important aspect in the context of the encoding and consolidation of new information is the expression of immediate early genes like arc, homer1a, c-fos, or egr1/zif-268. Following induction of so-called rapid primary response genes (Tullai et al., 2007; Saha et al., 2011; Saha and Dudek, 2013), mRNA expression and subsequent protein synthesis, plasticity-related proteins (PrPs) directly or indirectly impact synaptic structure and function. Interestingly, mRNA of PrPs can either be translated in the soma or locally in dendrites and synapses. The capture of mRNA or PrPs within the dendritic periphery and specifically within previously stimulated synapses is most likely realized through short-lived molecular tags (e.g., phosphorylated CAMKIIa, PKA, or CAMKIIb) as it was first suggested with the synaptic tagging and capturing hypothesis (Frey and Morris, 1997; Martin and Kosik, 2002; Bramham and Wells, 2007; Redondo and Morris, 2011). PrPs will then either support plasticity directly within the respective spine (effector proteins) or they will initiate the activation of secondary response genes (transcription factors), with subsequent transcription and protein synthesis.

#### Consolidation During Sleep

Several attempts have been made to identify underlying mechanisms for sleep-promoted consolidation of long-term memory. The “synaptic homeostasis hypothesis” (Tononi and Cirelli, 2006, 2014) considers sleep as an opportune time without interference from external stimuli and postulates that sleep is most important to ensure the brain’s energy balance and to maintain synaptic plasticity. Both aspects are presumably achieved through global synaptic downscaling, though the hypothesis concedes that large, recently potentiated synapses might be excluded from downscaling. In this scenario, sleep would benefit memory consolidation chiefly through a reduction in signal-to-noise ratio (de Vivo et al., 2017; Tononi and Cirelli, 2020). The “sequential hypothesis” (Giuditta et al., 1995; Giuditta, 2014) suggests that NREM sleep employs selective processes to weaken irrelevant or competing non-adaptive memories, while REM sleep preserves the remaining memories to integrate them with preexisting memories. In a similar way, it was proposed that transient neuronal changes, induced during waking, would change neuronal CREB-dependent excitability and prime (tag) synaptic clusters within neuronal ensembles and circuits (Seibt and Frank, 2019). Plasticity-related genes (i.e., mRNAs of IEGs) would be transcribed and targeted towards dendrites, thus being ready to be captured by spines upon reactivation for further potentiation or depression during NREM. REM sleep would then stabilize structural plasticity through protein-synthesis-dependent processes of synaptic strengthening/weakening, pruning as well as synaptogenesis (Seibt and Frank, 2012, 2019; Seibt et al., 2012; Sigl-Glockner and Seibt, 2019). REM sleep is indeed considered to be an opportune time for bidirectional synaptic change and refinement (Poe et al., 2010; MacDonald and Cote, 2021) as relatively high cholinergic activity (Marrosu et al., 1995) would favor induction of LTP (Hasselmo and Bower, 1993) while low norepinephrinergic activity (Aston-Jones and Bloom, 1981) would be essential for depression (Thomas et al., 1996; Katsuki et al., 1997). Additionally, theta activity of REM sleep has been proposed to promote selective strengthening and weakening of memories (Poe et al., 2000; Grosmark et al., 2012).

Ribeiro et al. (2007), pondering hippocampal disengagement and cortical engagement of spatial and episodic memories over time (Squire et al., 1993; Izquierdo and Medina, 1997; Frankland and Bontempi, 2005), propose that the repetition of wake-sleep cycles would produce the propagation of memories from the hippocampus to the cortex (corticalization). This is presumably accomplished with the two main sleep phases complementing each other functionally through hippocampal-cortical reactivation during SWS (Pavlides and Winson, 1989; Wilson and McNaughton, 1994; Peigneux et al., 2004; Ribeiro et al., 2004) and gene transcription dependent long-term memory storage during REM sleep (Ribeiro and Nicolelis, 2004; Ribeiro et al., 2004). In support of this notion, spatiotemporally distinct waves of enhanced mRNA expression of IEGs, indicative of increased synaptic activity, were detected after *in vivo* hippocampal LTP induction during wake and then during REM phases, first appearing in hippocampal areas, later in areas of the neocortex, each of them terminated in the following NREM sleep phase. With REM sleep promoting the transcriptional upregulation in the cortex, but not in the hippocampus, it was suggested to be an opportune window for hippocampus driven cortical activation, to play an instructive role in the communication of memory traces from the hippocampus to the cortex and being important for the stabilization of memory traces within cortico-cortical connections (Ribeiro et al., 1999, 2002, 2007; Almeida-Filho et al., 2018).

Finally, the “active systems consolidation hypothesis” (Diekelmann and Born, 2010; Klinzing et al., 2019; Feld and Born, 2020) incorporates the above-mentioned models and proposes, mainly based on studies concerned with declarative memories, that despite or in parallel to global synaptic downscaling, sleep promotes long-term memory through repeating cycles of sleep-phase dependent, systemic and synaptic consolidation processes. Systemic consolidation would rely on much shorter periods of synaptic consolidation as a subroutine (Dudai, 2012) for stabilizing engram representations in local networks over a time course of hours, days, or even years. During sleep, particularly during NREM sleep and driven by the precise timing of certain brain oscillations, here namely cortical slow oscillations (SO <1 Hz), thalamic spindles (12–15 Hz), and hippocampal sharp wave ripples (ripples 80–220 Hz, SWR: extracellular negative wave followed by fast ripple), previously encoded information would be “replayed”. This means neuronal and synaptic ensembles, that presumably comprise an engram, would be reactivated, in a sequentially true although time condensed activity pattern (Wilson and McNaughton, 1994; Kudrimoti et al., 1999; Nadasdy et al., 1999; Diba and Buzsaki, 2007; Euston et al., 2007; Ji and Wilson, 2007; Lansink et al., 2008; O’Neill et al., 2010; Clawson et al., 2021). In other words, several neurons, supposedly part of an engram, that fire in a particular temporal order during a behavioral task would be found to fire in the same order again during subsequent NREM sleep when SWRs occur. However, although the sequence of firing would be the same as during wake, the duration for one such sequence to complete would be shorter [e.g., several-fold in the hippocampus in accordance with an increased discharge probability of pyramidal neurons during SWR bursts (Csicsvari et al., 1999)], which was suggested to facilitate the likelihood of neuronal co-activation and thus Hebbian synaptic plasticity (Buzsaki, 1989; Atherton et al., 2015). Of note, replay in correlation with SWRs is also seen during awake-states (Foster and Wilson, 2006; Diba and Buzsaki, 2007; Karlsson and Frank, 2009), particularly during pauses in waking behavior (e.g., after running a track), and was interpreted to play a role in the evaluation of event sequences (reinforcement; Foster and Wilson, 2006). In cases of awake-replay after track running, the sequence of firing was found to be in reversed order (Foster and Wilson, 2006; Diba and Buzsaki, 2007).

**Figure 1:**
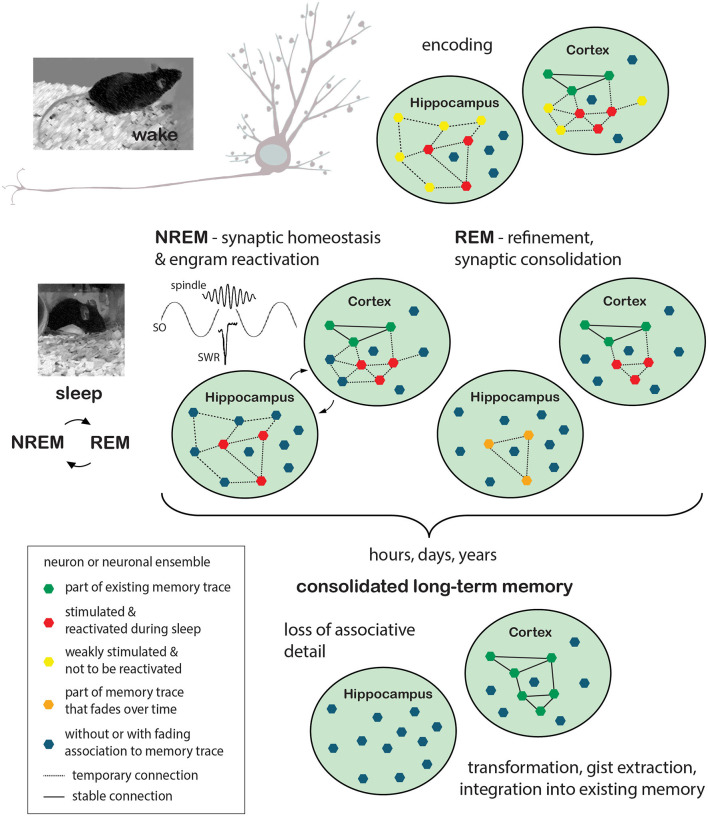
Memory formation: encoding during wake and consolidation during sleep. Encoding of new information takes place while the animal is awake and perceptive to sensory stimulation. Events are encoded in synchronized neuronal ensembles in a set of synapses that are stimulated in close temporal proximity. Induction of synaptic plasticity processes results in either transient or stable changes of synaptic strength and neuronal connectivity. Depending on the type of information, different brain regions are recruited during the encoding process. In the example given here, neuronal ensembles to encode the event (red polygons) are stimulated in the hippocampus as well as in cortical areas, with the former presumably responsible for more transient (orange polygons) preservation of associative details and the latter already containing a plethora of neuronal ensembles that represent the memory of previous events (existing memory; green polygons). Other neurons or neuronal ensembles, only weakly stimulated or entirely unrelated to the exemplified memory traces, are depicted in yellow and blue, respectively. Sleep is thought to benefit the maintenance of synaptic plasticity and the consolidation of long-term memory through synaptic homeostasis processes that lead to global synaptic downscaling in synergy with repeating cycles of sleep-phase dependent, systemic and synaptic consolidation processes. During NREM sleep, driven by the precise timing of cortical slow oscillations (SO), thalamic spindles, and hippocampal sharp wave ripples (SWR), previously encoded information would be “replayed”, i.e., previously stimulated neuronal ensembles (red polygons), as such tagged for maintenance, would be reactivated. In this scenario, the hippocampus is believed to support cortical long-term memory storage by driving cortical activation and serving as an associative hub among cortico-cortico connections. Repeated replay would subsequently be followed by the induction of plasticity-relevant gene expression and protein synthesis dependent scaling processes, presumably implemented during REM sleep and ultimately leading to the selective refinement of memory traces (orange polygons and dashed lines indicate temporary memory traces, solid lines indicate stable connections). The process of long-term memory consolidation can take any time from hours to years. During this time hippocampal involvement would lessen with memory traces dissipating and associative detail of the event thus getting lost. The cortical representations on the other hand would be stabilized and integrated into the network of pre-existing memory representations. Without the representation of associative detail, this information would however be “transformed” in a way, merely representing the gist of the experienced event. REM, rapid eye movement; NREM, Non-rapid eye movement.

Reactivation of neuronal ensembles and replay during NREM sleep was shown to support memory consolidation and thus later performance in memory retrieval (Rasch et al., 2007; Rudoy et al., 2009; Barnes and Wilson, 2014; Euston and Steenland, 2014; Yang et al., 2014; Ramanathan et al., 2015; Kim et al., 2019). In addition, it was demonstrated that inhibiting the reactivation of a subset of engram neurons during sleep can also transform the information that is stored, i.e., inhibiting the reactivation of sensory engram neurons during sleep after a preceding sensory cued fear conditioning task leads to a generalized fear response during later retrieval that was devoid of its associative aspect (Clawson et al., 2021). Repeated replay (possibly serving as tagging mechanism) would eventually be followed by the induction of plasticity-relevant gene expression during REM sleep and protein synthesis dependent scaling processes. The latter is presumably also implemented in periods of REM sleep, at high cholinergic activity, to selectively strengthen or weaken those synapses that were tagged for further processing. Temporally organized replay in different brain regions and minimal cholinergic activity during NREM are proposed to drive—at least for some types of memory—“redistribution” of encoded information from transient to permanent storage locations (e.g., for declarative memory from the hippocampus to cortical areas). While ensembles within the permanent storage location (cortex) are strengthened over time, engram representations within the transient (e.g., hippocampal) storage location would slowly decrease due to synaptic downscaling. Staying with the hippocampal-cortical example of episodic memories, progressing independence from the hippocampus, which is associated with storing context associations and thus details of episodic memory, would lead to “transformed” more abstracted, gist like memory representations (Figure 1).

## Homeostatic Plasticity in Support of Memory Consolidation During Sleep

Sleep is indisputably crucial for homeostatic regulation (Tononi and Cirelli, 2006, 2014), which relies on the synergistic action of slower feedback mechanisms, like global (Turrigiano et al., 1998; Turrigiano, 2008), and local (Sutton et al., 2006; Branco et al., 2008; Hou et al., 2008; Yu and Goda, 2009), synaptic scaling, or the regulation of intrinsic excitability (Zhang and Linden, 2003; Frick and Johnston, 2005; Niethard et al., 2017), in order to maintain stable function while at the same time preserving the specificity of synaptic changes that encode information (Turrigiano, 2012, 2017).

**Figure 2:**
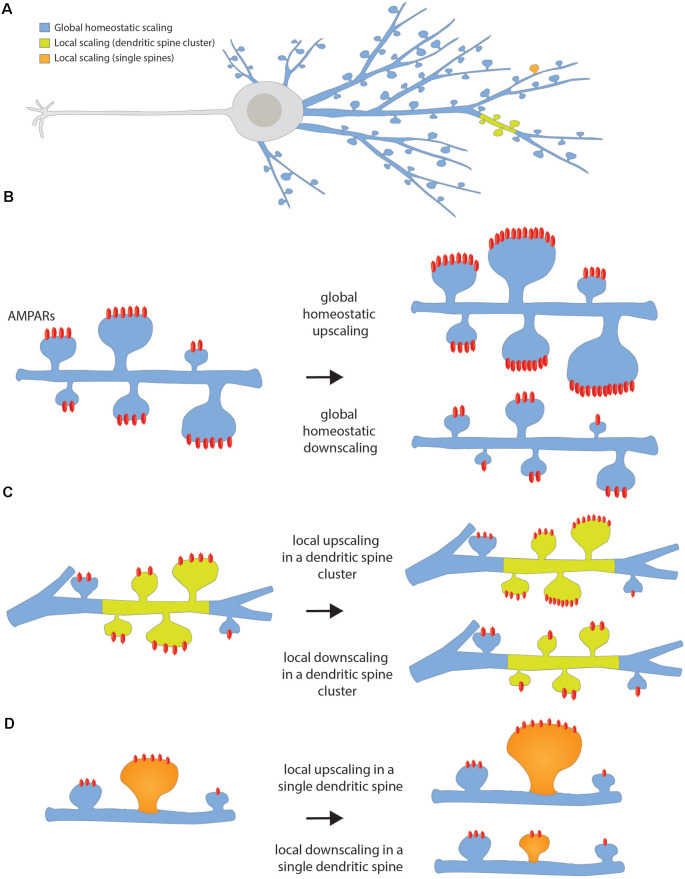
Homeostatic synaptic scaling. **(A)** Scheme of the whole neuron showing the color code for the different types of homeostatic scaling depending on the extent of the changes. **(B)** Global homeostatic up- or downscaling happens at the network level after a period of lowered or elevated firing rate, respectively. The neuron senses its own firing rate and it increases or decreases the strength of all its dendritic spines in a multiplicative manner, in such a way that the differences between synaptic weights are preserved. **(C)** Local up- or downscaling at the level of dendritic segments happens when a group of neighbor spines forming a cluster undergoes an increase or decrease in synaptic strength independently of the spines located in the neighbor dendritic segment. **(D)** Local up- or downscaling can also happen at the level of single dendritic spines when individual spines are able to autonomously sense their level of activity and compensate for it by increasing or decreasing its synaptic strength.

Synaptic scaling compensates for activity-dependent synaptic changes by maintaining the firing rate of any given neuron and neuronal circuit (Turrigiano, 2017) within a dynamic range around a set-point value (Turrigiano, 2008). In doing so, complete silencing or over-excitation and runaway potentiation, a self-reinforcing positive feedback loop following long-term potentiation (LTP) induction (Watt and Desai, 2010), are avoided. As synaptic drive increases and firing rate rises above the target level, surface expression of α-amino-3-hydroxy-5-methyl-4-isoxazolepropionic acid receptors (AMPARs) is decreased, thus reducing (downscaling) the strength of all inputs (Turrigiano, 2012, 2017), as the number of AMPARs on the surface is a direct correlate of synaptic strength. Upscaling on the other hand, after a period of silencing, increases synaptic strength and spine size (Shepherd and Huganir, 2007; Diering and Huganir, 2018). Global synaptic scaling applies this mechanism in a multiplicative way for all synapses of a neuron. Local synaptic scaling was proposed to work in a multiplicative way as well, although here, the functional unit would be a dendritic compartment of neighboring synapses rather than the whole neuron (Rabinowitch and Segev, 2008; Figure 2).

Homeostatic intrinsic plasticity regulation is based on a neuron’s ability to shift its excitability in an activity-dependent way by changing for instance the threshold for action potential firing. Neurons respond to low (or high) activity patterns by becoming more (or less) responsive to the input they receive (Karmarkar and Buonomano, 2006; Watt and Desai, 2010). The impact of intrinsic plasticity can be complex, involving not only alterations in gain or threshold, but also in spike frequency adaptation, afterpotentials, synaptic integration, local dendritic excitability, temporal firing patterns, and resonance characteristics (van Welie et al., 2004; Frick and Johnston, 2005; Trasande and Ramirez, 2007; Narayanan and Johnston, 2008; Watt and Desai, 2010).

Several studies reported global synaptic down-scaling and on an average decrease in neuronal excitability across sleep (Tononi and Cirelli, 2006, 2014; Vyazovskiy et al., 2009; Bushey et al., 2011; Maret et al., 2011; Grosmark et al., 2012; Yang and Gan, 2012; Miyawaki and Diba, 2016; de Vivo et al., 2017) yet subsets of neurons also maintain or even increase in spine number, synaptic strength, and firing rate during sleep(Ribeiro et al., 2007; Aton et al., 2014; Yang et al., 2014; Li et al., 2017; Niethard et al., 2017; Clawson et al., 2018; Raven et al., 2019).

### Structural Evidence of Plasticity During Sleep

The vast majority of studies delineate no changes in spine density, but a global decrease in spine size during sleep, presumably reflecting homeostatic downscaling. Thus, in cortical areas, spine numbers do not seem to differ between sleep and wake (Maret et al., 2011; de Vivo et al., 2017, 2019). However, two studies (Maret et al., 2011; Yang and Gan, 2012) examined spine turnover in the sensorimotor and somatosensory cortex of adolescent mice and observed higher spine elimination rates during sleep compared to natural wake or a period of sleep deprivation (SD). With regard to spine size, changes between wake and sleep, with an increase during wake and decrease during sleep, respectively, are reportedly age-dependent, either affecting large and small sized spines (pups; motor cortex; de Vivo et al., 2019), only small spines (adolescents; motor and somatosensory cortex; de Vivo et al., 2017) or only big spines (adults; motor cortex; Diering et al., 2017). Underlying causes for these differences might lie in different sleep-wake-patterns, a different status in the animals’ cortical and cognitive development, and with those different requirements on structural plasticity.

Somewhat conflicting results have been described for the hippocampus (mostly focused on Cornu Ammonis 1, CA1), as some studies found a decrease in spine number after SD compared to a period of undisturbed sleep (Havekes et al., 2016; Raven et al., 2019; Wong et al., 2019), while others reported that spine number (Ikeda et al., 2015; Spano et al., 2019; Gisabella et al., 2020) and size (Norimoto et al., 2018; Spano et al., 2019; Gisabella et al., 2020) decreased following periods of sleep.

The relevance of such processes was underscored in experiments where mice were challenged prior to sleep with a motor task and changes were analyzed using transcranial 2-photon-imaging of the motor cortex. Subsequent REM sleep appeared to contribute to memory consolidation by the refinement of learning-induced new synaptic connections (Li et al., 2017). A subset of newly formed spines was strengthened and maintained during sleep, while other spines were selectively eliminated (Li et al., 2017), and even new spine formation was reported during sleep after mice had performed a motor task (Yang et al., 2014). It was also shown that spine elimination during REM sleep in response to monocular deprivation or after fear conditioning would go along with reduced neuronal activity and was eliminated by blockade of Ca^2+^-spikes (Zhou et al., 2020).

### Electrophysiological Evidence of Plasticity During Sleep

How are structural changes in spine size across the wake and sleep cycle related to electrophysiological measures of synaptic efficacy? Several studies showed that the slope, amplitude, frequency, and synchrony of miniature excitatory postsynaptic currents (mEPSCs) and local field potentials (LFP) increase with wakefulness and decrease overall during sleep (e.g., Vyazovskiy et al., 2008, 2009; Liu et al., 2010; Gonzalez-Rueda et al., 2018; Norimoto et al., 2018). This is notwithstanding a recent study observing neuronal firing rates to increase in the neocortex and to decrease in CA1 during REM sleep or to diverge during REM and to homogenize during NREM sleep (Miyawaki et al., 2019). Slope and amplitude were found to correlate with changes in slow-wave activity, a marker of sleep pressure (Vyazovskiy et al., 2008, 2009; Liu et al., 2010). In addition, cortical LTP and LTP-like plasticity are induced more easily by tetanic stimulation after sleep (Vyazovskiy et al., 2008; Kuhn et al., 2016). The reduced threshold to induce LTP indirectly supports the notion of a net increase in synaptic strength after periods of waking and a net synaptic depression (homeostatic downscaling) after periods of sleep.

What are underlying mechanisms? Changes in mEPSC frequency are thought to result from modification of the presynaptic component of synaptic transmission, while amplitude changes indicate alterations in the postsynaptic component (Ungless et al., 2001). Whole-cell patch-clamp recordings from pyramidal neurons in acute slices of the somatosensory cortex of rats showed that the number of Ca^2+^-permeable AMPARs is reduced after NREM sleep compared to wake (Lante et al., 2011) lessening synaptic weights and thus presumably resetting cortical connections for subsequent plasticity induction (Lante et al., 2011). *In vivo* whole-cell recordings and optogenetic stimulation of presynaptic inputs in the cortex of adolescent mice during SWS-like activity revealed that stimulation during Down states of SWS activity leads to conventional spike timing-dependent plasticity (STDP), while Up states were found to be generally biased toward depression, i.e., decrease in excitatory postsynaptic potential (EPSP) slopes (Gonzalez-Rueda et al., 2018). During the Up states of SWS activity, only presynaptic stimulation that contributes to postsynaptic spiking would protect (yet not strengthen) the respective connections (Gonzalez-Rueda et al., 2018). However, further mechanisms by which sleep could support long-term memory consolidation might be related to the circuit activity that promotes the reactivation of specific neuronal ensembles during SWS (Kruskal et al., 2013). Memory relevant engram cells seem to be exempt from the overall decrease in firing patterns during sleep (Norimoto et al., 2018). Evoked potential responses in the somatosensory cortex of awake, adult cats were enhanced after an episode of slow-wave sleep as compared to an episode of wake (Chauvette et al., 2012). Subsequent *in vitro* studies showed that this enhancement is mediated by a postsynaptic mechanism that is calcium-dependent and requires hyperpolarization periods (slow waves) as well as the co-activation of both AMPA and NMDA receptors (Chauvette et al., 2012) as would be expected by reactivation of neuronal ensembles.

Analysis of firing rate homeostasis (FRH) in the context of monocular deprivation in the visual cortex of rats revealed that the typical shift in visual responses toward the non-deprived eye, that is seen after monocular deprivation (MD), is also sleep-dependent (Frank et al., 2001) as the activity of particular neurons was potentiated during sleep, both in NREM and REM phases, but not during wake, and this potentiation was prevented by sleep deprivation (Durkin and Aton, 2016). The assumption that replay might be part of the underlying mechanism in this was supported by the observation of increased neuronal activity in V1 during post-MD sleep (Aton et al., 2009). Potentiation was found to be dependent on NMDAR and PKA activity and involved phosphorylation events associated with LTP, indicating that synaptic strengthening *via* NMDAR and PKA activity would be a key step in sleep-dependent consolidation of ocular dominance plasticity (Aton et al., 2009). Hebbian depression mechanisms that are induced by MD, occurred during both, wake and sleep (Hengen et al., 2016). Re-opening of the eye (ER) would then be followed by a firing rate overshoot, consistent with Hebbian potentiation (Torrado Pacheco et al., 2020). Both, depression after MD and potentiation after ER, would subsequently be compensated by homeostatic processes, which, however, occurred in different arousal states: Downward homeostatic regulation was restricted to sleep (Torrado Pacheco et al., 2020), whereas upward homeostatic regulation only occurred during wake (Hengen et al., 2016).

### Sleep Deprivation and Synaptic Plasticity

What are molecular mechanisms underlying LTP impairments caused by the lack of sleep (Havekes et al., 2016)? In the CA1 area of adolescent mice cAMP- and PKA-dependent LTP is impaired in brain slices from animals that had been sleep deprived for 5 h (Vecsey et al., 2009). These changes were accompanied by decreased cAMP and phosphorylated cAMP response element-binding protein (CREB) at S133 levels and increased PDE4 levels after sleep deprivation in the brain of adult mice. 3 hr of SD significantly impaired LTP in the CA1 area in brain slices and also spatial memory if sleep deprivation was imposed 1 h after training (Prince et al., 2014). In contrast, if animals were sleep deprived immediately after training, LTP and spatial memory were not affected (Prince et al., 2014). These findings define a 3-h critical period, extending from 1 to 4 h after training, during which sleep deprivation impairs hippocampal function. In a recent study, it was shown that SD impairs synaptic tagging and capture (STC) in the hippocampus and behavioral tagging, two major mechanisms of associative learning and memory (Wong et al., 2019). Of note, SD impairs late- but not early-LTP (Kopp et al., 2006; Vecsey et al., 2009; Havekes et al., 2016).

## Molecular Mechanisms in Support of Memory Consolidation

The distinct roles of wakefulness and sleep in memory formation are reflected by molecular oscillations on the level of gene expression and the activity of kinases. Below, we will review the different cellular functions that can be deduced from these oscillations and how they affect synaptic plasticity over the 24-h cycle. This bird’s view perspective is complemented with a closer look at neuronal ensembles that are engaged in the encoding of information during wakefulness and that play a central role in memory consolidation. We discuss how neuronal activity during initial memory acquisition might “prime” these cells for extensive structural and synaptic remodeling in favor of highly activated connections during sleep. This process may strengthen and refine memory engrams, which would support the consolidation process on the network- and system-level.

### Gene Expression Patterns Across the Sleep-Wake Cycle

Systematic studies revealed that in the brain of mice around 6–8% of total transcripts “cycle” in expression across 24 h, with levels depending on the time of the day (Maret et al., 2007; Hor et al., 2019; Noya et al., 2019). These oscillations are especially pronounced at synapses. In synaptoneurosomal fractions of the forebrain, two-thirds of the synaptically enriched mRNAs, detected by deep sequencing, as well as around 12% of total synaptic proteins, detected by shot-gun proteomics were reported to cycle (Noya et al., 2019). Interestingly, the time-points of peak changes in gene expression for the majority of cycling transcripts and corresponding protein levels are found at the end of the wakefulness or sleep period.

**Figure 3:**
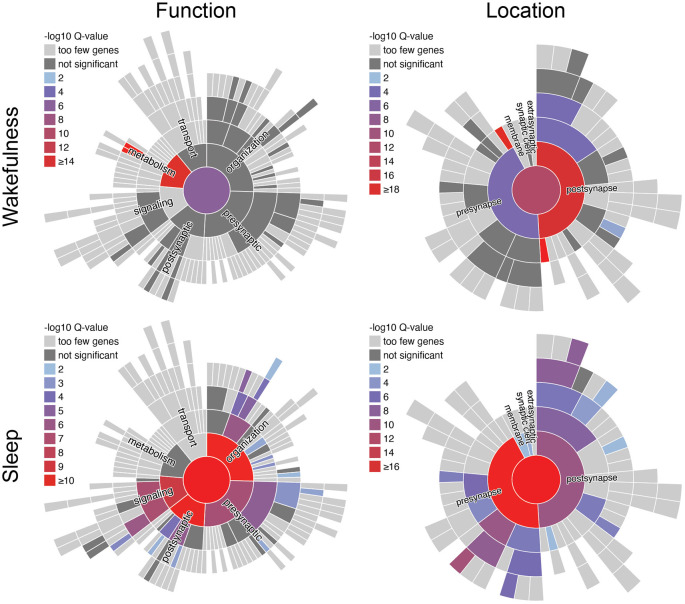
SynGO analysis of synaptically enriched transcripts from murine forebrain synaptoneurosomes from Noya et al. (2019). Functional terms of synaptically enriched transcripts with peak expression during wakefulness (ZT12–24) are enriched for metabolic processes, whereas during sleep (ZT0–12) terms regarding synaptic function and structure are enriched. SynGO (version: 20210225) annotations are based on published experimental evidence and were peer reviewed by experts (Koopmans et al., 2019). Function- and location-terms are hierarchically ordered, with the highest hierarchical term (“function at the synapse”/“synapse”) in the center. One-hundred and seventy out of 1,354 synaptically enriched genes with peak expression during wakefulness and 155 out of 728 synaptically enriched genes with peak expression during sleep, respectively, were recognized and mapped to unique SynGO annotated genes. They were compared to the default background list provided by SynGO, which contains all genes expressed in the brain. Colors indicate the significance of enrichment compared to the background set. ZT, Zeitgeber time.

Based on the database SynGO (Koopmans et al., 2019), analysis of the synaptically enriched, cycling transcripts from Noya et al. (2019), which peak during wakefulness or during sleep, reveals distinct biological function (Figure 3). During wakefulness, there is a strong increase of transcripts that are enriched for the functional GO-term “metabolism”, and here in particular “translation machinery at pre-/post-synapse”. Thus, many of these transcripts code for ribosomal proteins that show high expression levels throughout wakefulness reaching a maximum before sleep and then relatively quickly decay to minimal expression during sleep (Noya et al., 2019). While it has been shown that protein translation in the brain is important for memory formation during wakefulness and sleep (Tudor et al., 2016; Raven et al., 2020), the elevated expression of ribosomal proteins towards the end of wakefulness might be related to memory acquisition and an increasing need for memory consolidation, which is dependent on the synthesis of new proteins (Kelleher et al., 2004). In contrast, during sleep and the first hour of wakefulness increased expression of transcripts that are enriched for the functional GO-terms “synapse organization” (i.e., regulation of presynapse assembly, postsynaptic density assembly, regulation of synapse organization, synapse adhesion between pre- and post-synapse), “process in the postsynapse” (i.e., regulation of postsynaptic membrane neurotransmitter receptor levels, regulation of postsynaptic membrane potential), “synaptic signaling” (i.e., modulation of chemical synaptic transmission), and “process in the presynapse” (i.e., regulation of synaptic vesicle exocytosis, synaptic vesicle proton loading) is apparent. This indicates that during sleep the focus shifts towards mechanisms related to synaptic restructuring and refinement. With respect to memory formation, this may reflect the facilitation of consolidation during sleep, while external input is suppressed (Wang et al., 2005; Rawashdeh et al., 2007; Michel and Lyons, 2014; Levy et al., 2016).

### Oscillating Kinase Activity Facilitates Increased Synaptic Potentiation During Wakefulness and Depression During Sleep

Substantial oscillations across the wake-sleep cycle were also reported for post-translational modifications (Diering et al., 2017; Wang et al., 2018; Bruning et al., 2019). Phosphorylation sites in around 50% of detected synaptic phosphoproteins were shown to significantly cycle corresponding to the time of the day and independently of expression levels of the respective proteins (Bruning et al., 2019). Cycling phosphorylation peaks were found to cluster, similar to what could be observed for cycling mRNAs and proteins, either at the end of the wakefulness/transition to sleep phase or at the end of sleep/the beginning of wakefulness phase.

While oscillatory patterns were less apparent among phosphatases, around half of all 128 identified synaptic kinases contained at least one cycling phosphorylation site, indicative for cycling activity (Bruning et al., 2019). Identification of the kinases which were most active in either of the two clusters *via* motif and interaction analysis of the phosphoproteome, revealed two contrary landscapes with respect to synaptic plasticity. The kinases that were most active at the transition to or during early wakefulness, are prominent mediators of synaptic potentiation: CamKII, PKA, and PKC (Vyazovskiy et al., 2008; Cui et al., 2016; Diering et al., 2017; Bruning et al., 2019). Phosphorylation profiles of substrates of CamKIIα and PKC suggest that the activity of these kinases slowly builds up before it peaks and stays at a high level throughout wakefulness until it rapidly drops before sleep and then slowly builds up again (Bruning et al., 2019). On the other hand, kinases that had their most active period at the transition to or in early sleep are mostly associated with synaptic depression and homeostatic downscaling: Abl2, DCLK2, CDK5, and GSK3β (Diering et al., 2017; Bruning et al., 2019; see Table 1). The activity of GSK3β was shown to be high throughout sleep, before it rapidly drops at the transition to wakefulness, as indicated by the phosphorylation profile of one of its substrates (Bruning et al., 2019).

**Table 1:** The effects of the kinases CDK5, GSK-3β, Abl2, and DCLK2 on synaptic plasticity and evidence for their action during sleep.

Kinase	Where	Mechanism/Observation	Outcome	Publication	Does it happen during sleep?
CDK5	Post-synapse	Phosphorylation of PSD95 at Thr19, Ser25 and Ser35, which reduces multimerization of PSD scaffold and reduced clustering of ion channels	Reduced post synaptic currents	Morabito et al. (2004)	PSD95 Thr19 and Ser25 peak at ZT11 (Bruning et al., 2019)
		Phosphorylation of SPAR at Ser1328, which causes further phosphorylation through PLK2, leads to degradation of SPAR. This decreases SPAR-mediated recruitment of PSD95 to the post-synapse	Less recruitment of PSD95 to the post-synapse by SPAR	Pak and Sheng (2003) and Seeburg et al. (2008)	
		Induces interaction between GluN2B and calpain, leading to their degradation	Reduction of surface GluN2B levels and currents	Hawasli et al. (2007)	
		Enhances endocytosis of GluN2B-containing NMDAR	Reduction of GluN2B-containing NMDAR	Zhang et al. (2008)	
	Pre-synapse	Phosphorylation of synapsin I at Ser549 and Ser551, which increases binding of synapsin I to F-actin and sequesters SV in the resting pool	Reduced NT release	Verstegen et al. (2014)	Synapsin I Ser551 peaks at ZT0 (Bruning et al., 2019)
		Phosphorylation of dynamin I at Ser774, which allows phosphorylation of Ser778 by GSK-3β and enables activity-dependent bulk endocytosis of SV	Increased capacity to release NT	Clayton et al. (2010)	Dynamitin I S774 and S778 peak at ZT3–4 (Bruning et al., 2019)
GSK-3β		GSK-3β can be phosphorylated at Ser9 by kinases involved in LTP, including CamKII, PKA and PKC	Inhibition of GSK-3β activity	Sutherland et al. (1993), Li et al. (2000), Ballou et al. (2001), Song et al. (2010), Bradley et al. (2012), and Jaworski et al. (2019)	Phosphorylation of GSK-3β at Ser9 is minimal during sleep (Vyazovskiy et al., 2008, Bruning et al., 2019)
		Dephosphorylation of GSK-3β at Ser9 is mediated by PP1, following induction of NMDAR-LTD	Activation of GSK-3β	Szatmari et al. (2005) and Peineau et al. (2007)
	Post-synapse	Phosphorylation of KLC2 at residues 601–622, which enhances AMPAR internalization	Reduction of AMPAR currents	Du et al. (2010)	KLC2 Ser606 peaks at ZT5 (Bruning et al., 2019)
		Phosphorylation of PSD95 at Thr19	Reduced PSD95 levels, reduction of surface GluA1 in NMDAR dependent LTD	Szatmari et al. (2005), Peineau et al. (2007), and Nelson et al. (2013)	PSD95 Thr19 and Ser25 peak at ZT11 (Bruning et al., 2019)
	Inhibitory synapses	Phosphorylation of gephryn at Ser270, which leads to disintegration of inhibitory post-synapses and GABA_A_ receptor internalization	Reduced inhibitory currents	Tyagarajan et al. (2011) and Battaglia et al. (2018)	Gephryn Ser270 peaks at ZT2 (Bruning et al., 2019)
	Pre-synapse	Phosphorylation of P/Q type Ca2+ channels, leading to reduced presynaptic Ca^2+^ levels and formation of the SNARE complex	Reduced NT release	Zhu et al. (2010)	
		Overexpression reduces expression of synapsin I	Reduced NT release	Zhu et al. (2007)	
		Phosphorylation of dynamin I at Ser778, following phosphorylation of Ser774 through CDK5, which enables activity-dependent bulk endocytosis of SV	Increased capacity to release NT	Clayton et al. (2010)	Dynamitin I S774 and S778 peak at ZT3–4 (Bruning et al., 2019)
Abl2	Post-synapse	Binds to cortactin through phosphorylation of cortactin at S421 and S466, leading to spine stabilization and protection from severing through cofilin	Stabilization of spines during LTP	Courtemanche et al. (2015), Mikhaylova et al. (2018), and Shaw et al. (2021)	
		Phosphorylation of p190RhoGAP at Tyr1105, which reduces RhoA activity	Regulation of actin remodelling during structural plasticity	Hernandez et al. (2004) and Sfakianos et al. (2007)	p190RhoGAP Tyr1105 peaks at ZT11, 5 (Bruning et al., 2019)
DCLK1	Post-synapse	Isoform DCLK1-L binds to PSD proteins and reduces PSD95 and homer1 in spines	Reduction of post-synaptic PSD95 and homer1 levels and reduced surface GluA2	Shin et al. (2013)	
	Pre-synapse	Reduced vGLUT puncta at pre-synapse	Reduced SV release	Shin et al. (2013)	

These results show, that on a global scale, mechanisms related to synaptic potentiation prevail during wakefulness and synaptic depression-related mechanisms predominate during sleep (Vyazovskiy et al., 2008; Tononi and Cirelli, 2014). While this may cause a reduction of the average synaptic strength during sleep, as proposed in the “synaptic homeostasis hypothesis”, it does not exclude the occurrence of synaptic potentiation on a more local scale or generation of new spines (Yang et al., 2014; Diering et al., 2017; de Vivo et al., 2017). It is still a matter of debate if these effects are mainly driven by the physiological state of sleep in a sleep-homeostatic manner or by circadian effects that coincide with sleep (Michel and Lyons, 2014; Frank, 2016, 2021), but likely it is a combination of both. Notably, animals in the studies discussed above (Maret et al., 2007; Diering et al., 2017; Bruning et al., 2019; Hor et al., 2019; Noya et al., 2019) were not subjected to any cognitive tasks before sleep, which suggests that these mechanisms might be a default circuit property.

The accumulation of synaptic potentiation during wakefulness has been linked to an increase in sleep pressure in mammals and flies. Like in mammals, sleep in *Drosophila melanogaster* is regulated by circadian and homeostatic processes and has been shown to be important for memory consolidation following different learning tasks (Cirelli and Bushey, 2008; Donlea et al., 2009; Bushey et al., 2011; Dag et al., 2019; Donlea, 2019). Just like in mammals, consolidation was shown to depend on reactivation of neurons, that were involved in the acquisition of memory, during post-learning sleep (Dag et al., 2019), as well as on systems-level mechanisms, that can lead to “transfer” of the memory traces from one brain region to another over time (Cervantes-Sandoval et al., 2013; Dubnau and Chiang, 2013). Following learning, environmental enrichment, or forced wakefulness, flies show an increase in sleep pressure, accompanied by larger and more numerous synapses, which are reduced during subsequent sleep (Donlea et al., 2009; Donlea, 2019). Genetic upregulation of the synaptic strength through overexpression of the presynaptic scaffold protein *bruchpilot*, which drives plasticity of the active zone in *Drosophila*, was sufficient to promote sleep in a dosage- dependent manner in wildtype animals and different genetic models with reduced sleep (Huang et al., 2020). Conversely, knock-down of different active zone proteins, reducing synaptic strength, led to a reduction in sleep duration. Furthermore, the increase in phosphorylation of several synaptic proteins during wakefulness could be mimicked in genetic and pharmacological models of increased sleep pressure in mice (Wang et al., 2018). The activity of the MAP-kinase ERK, which is induced following synaptic potentiation and memory acquisition during wakefulness, was shown to promote sleep (Winder et al., 1999; Mikhail et al., 2017) and genetic deletion or inhibition of ERK in flies and rodents led to a reduction in total sleep duration (Vanderheyden et al., 2013; Mikhail et al., 2017).

### Enhanced Neuronal Activity and Learning During Wakefulness Prime Neurons for Processing During Sleep

Collectively, current evidence suggests that more synaptic connections get potentiated across wakefulness and the need for sleep increases, which promotes synaptic “renormalization” through depression and homeostatic scaling, as well as consolidation of memory during sleep (Tononi and Cirelli, 2014). Neurons adjust their transcriptional profile to enable plasticity and the encoding of memories, which is regulated by activity-induced transcription factors (TFs), such as pCREB, MEF2, and SRF (Yap and Greenberg, 2018). This is accompanied by epigenetic modifications that further promote the activity of these TFs. In whole brain samples both, spontaneous and forced wakefulness (sleep deprivation) cause a similar increase in chromatin accessibility at specific sites of the genome, which were correlated with enhanced, wakefulness-related gene expression (Hor et al., 2019). Furthermore, in cortical samples, SD was found to cause vast changes in the level of DNA methylation and hydroxymethylation in differentially expressed genes (Massart et al., 2014). Predicted upstream regulators that could profit from these changes and enhance the expression of their targets are mostly related to metabolism, synaptic transmission, and activity-dependent signaling (Massart et al., 2014).

The TF CREB has been shown to be crucial for memory formation (Lisman et al., 2018). It is well established, that increased activity of pCREB leads to an increase in the excitability of a neuron (Dong et al., 2006; Lopez de Armentia et al., 2007; Zhou et al., 2009; Yiu et al., 2014). This in turn induces a positive feedback loop that facilitates potentiation of spines. Neurons with high pCREB levels and increased excitability were shown to be more likely to become part of a memory engram (Han et al., 2007; Yiu et al., 2014; Park et al., 2016). Both, increased excitability and synaptic potentiation have been proposed to be mechanisms that underlie memory replay during sleep (Atherton et al., 2015; Lisman et al., 2018). Thus, the experience-related increase in neuronal activity in a subset of neurons that were engaged during previous wakefulness primes them through transcriptional adaptations that are accompanied by epigenetic modifications, as well as increased excitability and synaptic strength. Primed neuronal ensembles are subjected to replay and refinement during sleep.

### Immediate Early Genes Drive Plasticity and Are Critical for Memory Consolidation

Immediate early genes (IEGs) are among the downstream targets of the TFs pCREB, MEF2, and SRF (Yap and Greenberg, 2018). Characteristic for IEGs is their rapid and transient expression in response to stimulation, which does not, in contrast to “late-response” genes, require synthesis of new proteins. Many studies over the last decades have shown that they are involved in various forms of neuronal plasticity with a particularly important role in memory allocation and consolidation (Guzowski et al., 2000; Jones et al., 2001; Ramírez-Amaya et al., 2005; Plath et al., 2006; Bekinschtein et al., 2007, 2008; Ploski et al., 2008; Katche et al., 2010, 2012; Maddox and Schafe, 2011; Ren et al., 2014; Cao et al., 2015; Nakayama et al., 2015). IEGs that act in the nucleus as TFs, such as egr1/zif-268 or the AP-1 family member c-fos, have a complex effect on gene transcription and regulate the expression of many plasticity-related genes (Pérez-Cadahía et al., 2011; Duclot and Kabbaj, 2017). Since IEGs are induced following neuronal activity and memory acquisition, their expression levels were found to be elevated during wakefulness and reduced during sleep (Maret et al., 2007; da Costa Souza and Ribeiro, 2015; Noya et al., 2019).

Studies of egr1-deficient mice revealed that egr1 is required for memory consolidation, but not memory formation. While short-term memory of knock-out mice in different spatial and non-spatial learning tasks was intact, they failed when tested for long-term memory (Jones et al., 2001). Likewise, knock-out mice showed no impairments in early LTP in the dentate gyrus (DG) after tetanic stimulation of the perforant path, but the potentiation was lost 24–48 h later (Jones et al., 2001). The effects of egr1 on synaptic plasticity are mediated by its downstream effectors and one of them is the activity-regulated cytoskeleton-associated protein (Arc/Arg3.1; Li et al., 2005). Similar to the role of egr1 in memory consolidation, studies on arc-deficient mice could show that arc is not necessarily important for memory acquisition, but for the maintenance of long-term memory (Guzowski et al., 2000; Plath et al., 2006; Ploski et al., 2008; Maddox and Schafe, 2011; Ren et al., 2014; Cao et al., 2015). Originally, a role of arc in LTP maintenance was described (Guzowski et al., 2000; Chowdhury et al., 2006; Plath et al., 2006; Messaoudi et al., 2007), but this has been challenged by recent results (Kyrke-Smith et al., 2021). While its expression is a well-established indicator for neurons that are to become part of an engram (Guzowski and Worley, 2001; Ramírez-Amaya et al., 2005; Cao et al., 2015; Nakayama et al., 2015), arc localizes to dendritic spines (Link et al., 1995; Lyford et al., 1995; Chowdhury et al., 2006; Vazdarjanova et al., 2006; Zhang et al., 2015; Fernández et al., 2017) and the nucleus (Korb et al., 2013) to engage in various forms of plasticity, such as LTD (Plath et al., 2006; Park et al., 2008; Waung et al., 2008) and homeostatic plasticity (Shepherd et al., 2006; Gao et al., 2010; McCurry et al., 2010; Korb et al., 2013; El-Boustani et al., 2018). At the post-synapse, it mainly mediates a reduction in synaptic strength by endocytosis of AMPARs (Chowdhury et al., 2006; Rial Verde et al., 2006; Shepherd et al., 2006; Waung et al., 2008). Following its induction, arc is preferentially localized at inactive dendritic spines, suggesting a function as an inverse tag (Okuno et al., 2012, 2018), which is likely mediated through interaction with CamKIIβ. Corroborating its role as inverse tag and mediator of AMPAR-endocytosis, the synaptic content of arc was found to be negatively correlated with surface levels of AMPARs (Okuno et al., 2012). Even though, naïve mice show an overall reduction of arc expression during sleep (da Costa Souza and Ribeiro, 2015), it appears that sleep still influences the subcellular distribution of arc, to promote homeostatic plasticity (Korb et al., 2013; Honjoh et al., 2017). In the cortex, nuclear levels of arc were found to increase after 2 h of sleep (Honjoh et al., 2017) and high levels of arc expression in single neurons were negatively correlated with cytoplasmic GluA1 levels.

The IEG homer1a is another effector that acts at the synapse to modulate synaptic strength. Homer1a belongs to the homer family of synaptic scaffold proteins, which connect mGluR1/5 and their downstream effectors, the type-I inositol triphosphate receptors (IP3R) and PKC, with each other and couple them to the postsynaptic density (PSD; Sala et al., 2001, 2003). This allows mGluR1/5 and IP3R to act in concert, to promote the facilitation of NMDAR-mediated LTP (Lu et al., 1997; D’Antoni et al., 2014; Martin et al., 2019). The IEG homer1a can abolish these effects in a dominant negative fashion, by disrupting and disintegrating the homer scaffold complex (Tu et al., 1998; Kammermeier et al., 2000; Sala et al., 2003; D’Antoni et al., 2014; Diering et al., 2017; Martin et al., 2019). This leads to a reduction in synaptic NMDAR currents and of the LTP-promoting effects of GluR1/5 signaling (Lu et al., 1997; Bertaso et al., 2010; D’Antoni et al., 2014). Instead, intracellular binding of homer1a to mGluR1/5 induces an “alternative” form of mGluR1/5-signaling, which is constitutively active and agonist-independent (Ango et al., 2001; Martin et al., 2019). The exact identity of the alternative mGluR1/5 transduction pathway is not known, but it was shown to mediate homeostatic plasticity by increased internalization of synaptic GluA1 and -2 containing AMPAR through a reduction in tyrosine phosphorylation levels (Hu et al., 2010). Overexpression of homer1a in either wildtype or arc-deficient neurons causes a similar reduction of GluA1 and -2 surface levels, suggesting that the effects of homer1a and arc on synaptic strength are independent.

Interestingly, during sleep, homer1a is redistributed to the PSD. This redistribution is driven by circadian oscillations of neuromodulators (Diering et al., 2017). The elevated activation of noradrenaline-receptors during wakefulness actively prevents homer1a from entering dendritic spines, while the activation of adenosine receptors, a correlate of sleep need, leads to synaptic accumulation (Bjorness et al., 2016; Diering et al., 2017). Therefore, the homer scaffold complex is disintegrated during sleep, as evidenced by the reduced association of mGluR1/5, IP3R, and PKC with the PSD. This is accompanied by the reduction of total GluA1 and -2 levels in PSD-fractions and lower phosphorylation levels of GluA1—effects that are abolished in homer1a-deficient mice. On a behavioral level, this is reflected by the observation that wildtype mice, if treated with mGluR1/5 inhibitors during sleep, but not wake, show impaired memory consolidation after fear conditioning, reflected by an increase in fear generalization (Diering et al., 2017).

In summary, IEGs act on different levels to mediate synaptic plasticity and enable long-term memory consolidation. While IEGs like egr1 drive the expression of genes that are relevant for plasticity in general, others like arc and homer1a exert a direct effect on synaptic strength, mainly by reducing synaptic strength through LTD or homeostatic scaling. This may be of particular importance for neurons that were involved in memory formation during the day, as an increased load of information would require to be stored and consolidated or might have to be discarded. Homeostatic scaling and the function of arc as an inverse tag might serve to clear memory from noise, i.e., traces, that are potentially not relevant for long-term preservation, thus enabling ongoing storage of new memories in the long run.

### IEG Expression Increases in Primed Neurons During Sleep to Promote Long-Term Memory Consolidation

IEG expression is driven in a sleep-homeostatic fashion. It is enhanced by neuronal activity, peaks towards the end of wakefulness periods, and drops during sleep (Maret et al., 2007; da Costa Souza and Ribeiro, 2015; Noya et al., 2019). However, if animals are exposed to experiences that elicit memory encoding, IEG expression is found to be increased in a regionally specific manner during and dependent on subsequent REM sleep phases (Ribeiro et al., 1999, 2002, 2007; Calais et al., 2015). Also, studies in the developing visual cortex of cats showed an increase of IEG protein translation (but not transcription) during REM (not NREM) sleep following the induction of plasticity through monocular deprivation, which correlates with the sleep-dependent shift in visual responses toward the nondeprived eye (Seibt et al., 2012; Renouard et al., 2018).

Neurons that were involved in memory encoding during wakefulness are subjected to replay during SWS sleep (Wilson and McNaughton, 1994; Lee and Wilson, 2002; Lisman et al., 2018), likely primed by CREB-dependent increase in neuronal excitability and synaptic potentiation (Lisman et al., 2018). The reactivation of neuronal ensembles might then, in turn, promote the activation of CREB during sleep (even though it is not known if this might occur in NREM or REM) and facilitate the reinduction of IEGs during REM sleep. SWS embedded replay alone was found to be insufficient to upregulate IEG expression but required subsequent REM sleep (Ribeiro et al., 1999, 2002, 2007; Seibt et al., 2012; Calais et al., 2015; Renouard et al., 2018). Nevertheless, following exploration of novel objects, sleep spindle amplitudes that were recorded in the cortex of rats during NREM sleep showed a strong positive correlation with subsequent increase of IEG expression during REM sleep, suggesting a functional interplay of both sleep states (Ribeiro et al., 2007). It might be, that replay during SWS could act, similar to neuronal activation during awake encoding, as a mechanism to (re)select neuronal ensembles and tag synaptic connections for further processing during subsequent REM sleep (Almeida-Filho et al., 2018; Seibt and Frank, 2019). The underlying mechanisms that restrict the increase of IEG expression to periods of wakefulness and REM sleep are not known but may be related to shared features, such as increased levels of acetylcholine and high theta power.

Several studies, that did not specifically investigate sleep, could show that, following different hippocampus-dependent learning tasks and an initial induction of IEGs in mice, another wave of IEG upregulation occurs in hippocampal CA1 neurons several hours post-training (Ramírez-Amaya et al., 2005; Bekinschtein et al., 2007, 2008; Katche et al., 2010, 2012; Nakayama et al., 2015). Importantly, neurons that would express arc during the initial induction were found more likely to express arc also later on (Ramírez-Amaya et al., 2005; Marrone et al., 2008; Nakayama et al., 2015). Blocking the second rise of IEG expression through hippocampal injection of either egr1, c-fos, or arc antisense oligodeoxynucleotides, was found to result in impaired long-term memory performance 7 days after training, which was accompanied by reduced synaptic pruning in CA1 pyramidal cells, while short-term memory performance 2 days after training was intact (Katche et al., 2010, 2012; Nakayama et al., 2015). Similarly, animals that were genetically deficient for the respective IEGs showed impaired maintenance of memory, but no deficit during initial memory acquisition (Guzowski et al., 2000; Jones et al., 2001; Plath et al., 2006; Ploski et al., 2008; Maddox and Schafe, 2011; Ren et al., 2014; Cao et al., 2015).

**Figure 4:**
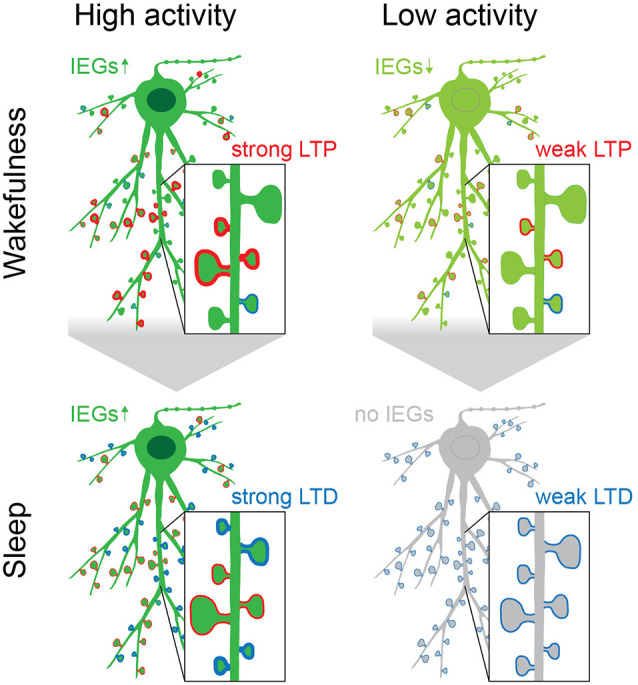
The acquisition of new memories during wakefulness causes upregulation of IEGs (green), which is followed by a second rise of IEG expression during sleep, preferentially in neurons that were highly active, exhibit increased intrinsic excitability and are constituents of initial memory engrams. The upregulation of IEGs during sleep is initiated during REM, following replay during NREM, and causes homeostatic plasticity (i.e., downscaling) to reduce noise and interferences from “old” or redundant memory traces (spines with blue rim). Spines that are reactivated during replay may be strengthened and protected from downscaling (spines with red rim; Rosanova and Ulrich, 2005; Chauvette et al., 2012; Kruskal et al., 2013; Yang et al., 2014; Atherton et al., 2015; Li et al., 2017; Lisman et al., 2018). This may happen in a spine- or dendrite-specific manner, as it has been shown that during sleep dendritic segments experience Ca^2+^-spikes that are separate from the cell body, as well as dendrite specific plasticity (Yang et al., 2014; Kastellakis et al., 2015; Li et al., 2017; Seibt et al., 2017). While the effects of IEGs during sleep may be most specific to neurons that are part of newly formed engrams (dark green neurons), the kinases GSK3β, Abl2, DCLK2, and CDK5 may act in concert to reduce synaptic strength on a global scale (Table 1, Diering et al., 2017; Bruning et al., 2019). Their combined action counterbalances the excess potentiation in the brain during wakefulness by “renormalizing” connections and thus aids the selective refinement of memory engrams (Cao et al., 2015; Attardo et al., 2018).

Collectively published data suggest that IEG-upregulation following initial memory acquisition occurs in waves with the second wave being of particular importance for long-term memory maintenance. In such a scenario one might speculate that memory replay during NREM and subsequent REM sleep could represent excellent candidates to drive this second wave of increased IEG expression. Accordingly, both sleep phases would be required to work synergistically to achieve long-term memory consolidation, even though the exact mechanism bridging the two is still not understood (Figure 4).

## Conclusions

It is nowadays firmly established that sleep is of central importance to maintain synaptic plasticity and to support memory consolidation with homeostatic scaling and Hebbian plasticity working in concert to reduce signal-to-noise ratio on a global level, yet maintaining or refining neuronal ensembles that comprise an engram. Less clear are the molecular mechanisms that contribute to long-term memory formation. Gene expression patterns and oscillating kinase activity across the wake-sleep cycle support distinct biological functions with increased synaptic potentiation during wakefulness and synaptic depression during sleep. Yet, enhanced neuronal activity during wakefulness also primes neurons for reactivation, supporting Hebbian plasticity as well as homeostatic scaling processes, and subsequent synaptic refinement during sleep. Immediate early genes mediate plasticity and are critical for memory consolidation. While IEGs like egr1 drive the expression of plasticity relevant genes in general, others like arc and homer1a exert direct effects, mainly reducing synaptic strength through LTD or homeostatic scaling. This molecular framework will set the stage for a more mechanistic understanding of memory consolidation and opens up new avenues to integrate findings at the systems level with the expression of plasticity at the cellular level as well as at the level of individual synapses.

## Glossary

**Engram**: persistent memory traces representing external or internal experiences of the brain.

**Activity-Dependent plasticity**: Stimulus-induced synapse-specific modification of synaptic strength and cell-specific changes of neuronal excitability.

**Hebbian plasticity**: An input-specific form of activity-dependent plasticity, consisting of a persistent enhancement (**long-term potentiation, LTP**) or decrease (**long-term depression, LTD**) of the synaptic transmission, yielding an increase or a decrease of synaptic weights, respectively.

**Homeostatic plasticity**: A mechanism that allows neurons to sense their own level of activity and to adjust their properties to maintain a stable function and avoid extremes of complete silence or over excitation.

• **Synaptic scaling**: Homeostatic plasticity mechanism working in a feedback manner that helps neurons to maintain stability. It responds to changes in the level of global synaptic efficacy and membrane excitability, allowing neuronal networks to maintain dynamic response properties. It maintains the firing rate of a given neuronal circuit within a dynamic range: as synaptic drive increases and firing rate rises above the target level, homeostatic mechanisms are engaged that reduce the strength of all inputs, that is, a downscaling process. During downscaling, surface expression of AMPAR decreases, and in turn synaptic strength is reduced. On the contrary, during upscaling after a period of silencing, synaptic strength and spine size will increase. Two types of scaling mechanisms have been described in neurons; **global homeostatic scaling**, in which the entire neuronal network is altered in a multiplicative way, thus the relative differences in synaptic weighs are preserved, and **input-specific synaptic scaling**, occurring at the level of a single dendritic branch (quasi-local) or even at single spines (local), without affecting neighboring synapses (Figure 2). Compared to Hebbian plasticity, scaling operates over a longer temporal scale (hours).

• **Synaptic pruning**: A mechanism used by neurons to maintain firing rate stability *via* the regulation of the number of synapses. This process is especially relevant during postnatal development when neural circuits are shaped by activity-dependent elimination of redundant synapses.

• **Regulation of intrinsic excitability**: Homeostatic plasticity mechanism that modulates the membrane properties of the postsynaptic neuron through the regulation upon the activity of the ion channels at the cell membrane. It modulates the sensitivity of the neuron thanks to activity-dependent alterations in the properties or levels of voltage-dependent Na^+^, Ca^2+^, Cl^–^ and K^+^ channels.

• **Excitation/Inhibition balance**: Dynamic adjustment in the relative strengths of excitatory and inhibitory feedback onto pyramidal neurons, which is an important component of firing rate homeostasis. Excitation and inhibition are regulated in opposite directions, and probably by independent mechanisms.

**Heterosynaptic plasticity**: A type of plasticity not limited to active synapses, but affecting neighboring synapses to the one receiving the stimuli. It may happen after episodes of strong postsynaptic activity and it does not show input specificity.

**Clustered plasticity**: Plasticity seems to be compartmentalized within a given dendritic segment, suggesting that clusters, rather than single synaptic contacts, may be a fundamental unit for storage of long-term memory. The spread of signaling molecules inside a dendritic segment, or the “synaptic tagging and capture” hypothesis might underlie clustered plasticity.

**Metaplasticity**: Mechanism by which neural activity at one time point alters cells or synapses in a way that it changes their ability to undergo LTP/LTD upon another activity event later in time, thus preventing the saturation of LTP and LTD. The term metaplasticity means plasticity of plasticity, as the previous history of activity at a certain synapse can modulate upcoming plasticity events by means of modifying the threshold for the induction of this following plasticity event.

**Silent synapses**: A type of glutamatergic excitatory synapses that possess NMDARs but lacks functional AMPARs, so it cannot mediate neurotransmission. Chronic activity blockade leads to the creation of new silent synapses, and they can be unsilenced by coordinated pre- and postsynaptic activity, such as in the case of an LTP induction protocol, as the activation of NMDARs promotes subsequent AMPARs recruitment to the postsynaptic membrane.

## Author Contributions

IR-R, SS, MRK, and AMO contributed to the conception and the design of this review. Each wrote individual sections of the manuscript. All authors contributed to the article and approved the submitted version.

## Conflict of Interest

The authors declare that the research was conducted in the absence of any commercial or financial relationships that could be construed as a potential conflict of interest.

## Publisher’s Note

All claims expressed in this article are solely those of the authors and do not necessarily represent those of their affiliated organizations, or those of the publisher, the editors and the reviewers. Any product that may be evaluated in this article, or claim that may be made by its manufacturer, is not guaranteed or endorsed by the publisher.

## Abbreviations

Abl2: Abelson-related gene
Arc/Arg3.1: Activity Regulated Cytoskeleton Associated Protein
AMPAR: α-amino-3-hydroxy-5-methyl-4-isoxazolepropionic acid receptor
CA1: Cornu Ammonis 1
CamKII: Calcium/calmodulin-dependent protein kinase type II
cAMP: Cyclic adenosine monophosphate
CDK5: Cyclin-dependent kinase 5
C-fos: Proto-oncogene c-Fos
CREB: cAMP response element-binding protein
DCLK1: Doublecortin like kinase 1
DG: Dentate gyrus
EEG: Electroencephalogram
Egr1/zif-268: Early growth response protein 1
EPSP: Excitatory postsynaptic potential
ERK: Extracellular signal-regulated kinase
FRH: Firing rate homeostasis
GABA_A_ receptor: Gamma-aminobutyric acid receptor type A
GluAx: Glutamate ionotropic receptor AMPA type subunit x
GluNx: Glutamate ionotropic receptor NMDA type subunit x
GSK3ß: Glycogen synthase 3β
Homer1: Homer protein homolog 1
IEG: Immediate early gene
IP3R: Inositol trisphosphate receptor
KO: Knock-out
LFP: Local field potential
LTD: Long-term depression
LTP: Long-term potentiation
MAP: Mitogen-activated protein
MEF2: Myocyte-specific enhancer factor 2
MD: Monocular deprivation
mEPSC: Miniature excitatory postsynaptic currents
mGluR1/5: Group I metabotropic glutamate receptors 1 and 5
MTL: Medial temporal lobe
NMDAR: N-methyl-D-aspartate (NMDA) receptor
NT: Neurotransmitter
NREM: Non-REM
RhoA: Ras homolog family member A
p190RhoGAP: A Rho family GTPase-activating protein
PDE4: Phosphodiesterase 4
PKA: Protein kinase A
PKC: Protein kinase C
PLK2: Polo-like kinase 2
PrPs: Plasticity-related proteins
PSD: Postsynaptic density
REM: Rapid eye movement
Serx: Serine residue at position x
SD: Sleep deprivation
SPAR: Spine-associated Rap GTPase activating protein
SNARE complex: SNAP receptor complex
STC: Synaptic tagging and capture
SO: Slow oscillations
SV: Synaptic vesicle
STDP: Spike timing-dependent plasticity
SWR: Sharp wave ripples
SWS: Slow-wave sleep
SRF: Serum response factor
TF: Transcription factor
Thrx: Threonine residue at position x
Tyrx: Tyrosine residue at position x
vGLUT: vesicular glutamate transporter
ZT: Zeitgeber time (ZT0 sleep/ZT12 wakefulness period onset).
